# Association between problematic internet use and depressive symptoms among individuals at high-risk of cannabis use disorder: a cross-sectional study in Quebec (Canada)

**DOI:** 10.1016/j.abrep.2026.100730

**Published:** 2026-07-29

**Authors:** Rasolofomamonjy Rasoamiadana Volanirina, Dufour Isabelle, Hudon Catherine, Carrier Nathalie, Muñoz Gómez Natalia, Brodeur Magaly

**Affiliations:** a Department of Family Medicine and Emergency Medicine, University of Sherbrooke, Faculty of Medicine and Health Sciences, 3001, 12e Avenue Nord, Sherbrooke J1H 5N4, Quebec, Canada; b School of Nursing, University of Sherbrooke, Faculty of Medicine and Health Sciences, 3001, 12e Avenue Nord, Sherbrooke J1H 5N4, Quebec, Canada; c Research Center on Aging, University of Sherbrooke, 1036 rue Belvédère Sud, Sherbrooke, QC, J1H 4C4, Canada; d CHUS Research Center, University of Sherbrooke, 3001, 12e Avenue Nord, Sherbrooke J1H 5N4, Quebec, Canada

**Keywords:** Problematic internet use, Cannabis, Addiction, Cannabis use disorder, Depressive symptoms, Quantitative

## Abstract

**Background:**

Cannabis use disorder affects a substantial proportion of the population and is often accompanied by mental health comorbidities, including depressive symptoms and problematic Internet use (PIU). This study explores the association between PIU and depressive symptoms among adults at high-risk of cannabis use disorder, a relationship insufficiently explored despite the high prevalence of these conditions in this population.

**Methods:**

We conducted a cross-sectional, correlational study using data from the "*Cannabis Use, Mental Health, and Problematic Internet Use in Quebec (Canada)*" study. Included were Quebec (Canada) adults at high-risk of cannabis use disorder. First, we described and compared the characteristics of participants with and without PIU; second, we used multivariable logistic regression to model the association between PIU and depressive symptoms while controlling for various sociodemographic and clinical factors.

**Results:**

Among 874 participants, 93 (10.6%) had PIU, and 232 (26.5%) reported depressive symptoms. Depressive symptoms were significantly more prevalent among those with PIU (58.1%) than among those without PIU (22.8%). The PIU group predominantly consisted of young adults, males, single persons, employed individuals, and those with higher incomes. This group also exhibited problematic use of alcohol and other substances and had a higher median score for anxiety symptoms. Our regression model showed that PIU was significantly associated with depressive symptoms (*OR* = 2.19 [1.15–4.19]; *p* = .018).

**Conclusion:**

Our findings emphasize an emerging public health concern and stress the need for further investigation into this association within the context of high-risk of cannabis use disorders in Quebec (Canada) and other sociocultural settings.

## Highlights

• This study included adults at high-risk of cannabis use disorder.
• Depressive symptoms are common among those with problematic Internet use (PIU).
• Other distinct sociodemographic and clinical patterns are seen in those with PIU.
• PIU is independently associated with depressive symptoms in this population.
• Quebec's sociocultural context should be considered in interpreting these findings.

## 1 Introduction

Cannabis ranks among the most widely used psychoactive substances globally, following alcohol and tobacco (Peacock et al., 2018). In 2022, an estimated 228 million people—representing 4.4% of the global population aged 15 to 65—used cannabis, with a higher prevalence in the Americas (11.4%) and Oceania (11.8%) (United Nations Office on Drugs and Crime, 2024). While cannabis use is illegal in several countries, its recreational use has drawn increasing attention in the public health and regulatory spheres (Bahji & Stephenson, 2019).

In Canada, the legalization of cannabis for recreational use came into effect in 2018 under the Cannabis Act. The Act aimed to keep cannabis out of the hands of youth, prevent profits from going to criminals, and protect public health and safety by providing legal access for adults (Government of Canada, 2018). Since then, the prevalence of cannabis use has risen: 22% of Canadians reported use in 2018, compared to 26% in 2024 (Health Canada, 2024). While most recreational use has no major consequences, a significant proportion progresses to problematic use, which can lead to cannabis use disorder (Conus & Davison, 2024; Leung et al., 2020).

Cannabis use disorder is defined in the Diagnostic and Statistical Manual of Mental Disorders (DSM-5) as a problematic pattern of cannabis use leading to clinically significant impairment or distress over a 12-month period (American Psychiatric Association, 2022). A key feature is continued use despite negative physical (e.g., respiratory issues), psychological (e.g., worsening mental health), or social (e.g., interpersonal conflicts) consequences (American Psychiatric Association, 2022). In 2016, approximately 22 million people worldwide were affected by cannabis use disorder (Degenhardt et al., 2018). People who use cannabis have a one-in-five risk of developing the disorder (Leung et al., 2020). In Canada in 2019–2020, 4.7% of past-year cannabis consumers aged 15 years or older were considered to experience impaired control and might have been at risk of cannabis use disorder (Statistics Canada, 2023a).

Cannabis use disorder frequently co-occurs with mental health disorders (Gorelick, 2018; Hasin et al., 2016). An increase in the severity of one disorder tends to intensify the symptoms of the other, suggesting a mutually reinforcing relationship (Hasin et al., 2016). Mental health disorders include other substance use disorders (e.g., alcohol, opioids), psychotic disorders, and mood disorders such as depression (Connor et al., 2014; Gorelick, 2018; Hasin et al., 2016; Kuhns et al., 2022).

Globally, approximately 4% of the population experienced depression in 2021 (World Health Organization, 2023). In Canada, the prevalence of major depressive episodes among those aged 15 and older increased from 4.7% in 2012 to 7.6% in 2022 (Statistics Canada, 2023b). Depression includes multiple diagnostic subtypes, each distinguished by its own set of symptoms (American Psychiatric Association, 2022). In this study, we broadly refer to depressive symptoms without specifying diagnostic categories to capture this diversity. Depressive symptoms encompass emotional, cognitive, and physical manifestations without necessarily meeting the criteria for a specific depressive disorder (American Psychiatric Association, 2022; Coryell, 2023; Kroenke et al., 2009; Martin et al., 2006). Symptoms include persistent low mood, loss of interest, sleep and appetite disturbances, poor concentration, excessive guilt, and, in severe cases, suicidal ideation or suicide (American Psychiatric Association, 2022; World Health Organization, 2023). Numerous factors are associated with depressive symptoms, including sociodemographic characteristics (e.g., age, gender, marital status), psychological factors (e.g., anxiety), substance use, and behavioral addictions, such as problematic Internet use (PIU) (Cai et al., 2023; Hua et al., 2025; Kumar et al., 2024; Zheng et al., 2023).

PIU is an emerging public health concern characterized by the inability to control Internet use despite its adverse impact on daily functioning. Its effects can be physical (e.g., fatigue), psychological (e.g., depressive symptoms), or social (e.g., isolation, conflict) (Block, 2008; Caplan, 2010; Davis, 2001; Spada, 2014). PIU is conceptualized as an umbrella construct encompassing diverse forms of problematic online behavior. The literature distinguishes between generalized PIU, reflecting an overall dysregulated pattern of Internet use across activities, and specific problematic Internet uses, such as gaming, social media use, or online gambling, which may involve distinct underlying mechanisms (Caplan, 2010; Davis, 2001). In the present study, PIU refers to a generalized pattern of excessive and uncontrolled Internet use, rather than engagement in a single specific online activity. A meta-analysis estimated the global prevalence of PIU to be 7% (Pan et al., 2020). A large cross-sectional study conducted in 2015 investigated PIU among adults from 15 countries, with Canadian data indicating a prevalence of 2.9% (Lopez-Fernandez et al., 2023).

A recent meta-analytic review conducted among students from diverse cultural and demographic backgrounds suggests a moderate positive association between PIU and depressive symptoms in more general population (Cai et al., 2023). Separately, research suggests that people at high-risk of cannabis use disorder may be exposed to PIU (Lanthier-Labonté et al., 2020) and that cannabis use disorder frequently co-occurs with depressive symptomatology (Hasin & Walsh, 2020; Kuhns et al., 2022; Onaemo et al., 2021; Sorkhou et al., 2024). Network analysis evidence also indicates links between depressive symptoms and technology-based addictive behaviors (including Internet use), as well as substance use (including cannabis) (Shmulewitz et al., 2024) — supporting potential co-occurrence of PIU and depressive symptoms in population at high-risk of cannabis use disorder. To our knowledge, no study has specifically evaluated the PIU–depressive symptoms association in adults at high-risk of cannabis use disorder, although this is a clinically relevant subgroup characterized by elevated rates of depressive symptoms and potentially PIU compared with the general population. By focusing on this higher-risk context, the present study extends earlier work by clarifying whether PIU is associated with depressive symptoms above and beyond the broader comorbidity burden typical of population at high-risk of cannabis use disorder. Understanding this association may inform the development of integrated prevention strategies, enhance screening practices by incorporating PIU and depressive symptoms alongside cannabis use disorder, and guide the implementation of targeted interventions in at-risk populations.

As a first study to examine PIU and depressive symptoms among individuals at high-risk of cannabis use disorder, this study aims to (1) describe the characteristics of individuals according to the presence or absence of PIU, including depressive symptoms, and (2) to model the association between PIU and depressive symptoms.

## 2 Methods

This cross-sectional descriptive correlational study (Fortin & Gagnon, 2022) is based on secondary data analysis from the project “Cannabis Use, Mental Health, and Problematic Internet Use in Quebec (Canada)”. The project is described elsewhere (Brodeur et al., 2024). The larger study received ethics approval by the Research Ethics Committee and Scientific Evaluation Committee of the *Centre intégré universitaire de santé et de services sociaux (CIUSSS) de l'Estrie—Centre hospitalier universitaire de Sherbrooke (CHUS)* on July 19, 2023 (reference number : 2024–5139-CyberD-Cannabis), and all participants provided informed consent including consent for secondary analyses. An amendment for secondary data use was submitted to and approved by the relevant Research Ethics Board.

### 2.1 Study participants and sampling

Participants for the larger research project were recruited online between April 18 and May 15, 2024. Eligibility criteria included being 18 years or older, residing in the province of Quebec (Canada), and having used cannabis for recreational purposes at least once per week during the past 12 months. A total of 1502 participants were recruited. For this secondary analysis, we focused specifically on individuals at high-risk of cannabis use disorder based on a Cannabis Abuse Screening Test (CAST) score ≥ 7 (Legleye et al., 2007), resulting in 874 participants included in the analysis.

### 2.2 Data collection procedure

Data were collected using a self-administered online questionnaire, available in French and English, and composed of validated instruments. A firm specializing in surveys and web panels conducted the survey using a stratified random sampling approach to ensure representativeness by age, sex, gender, language, and geographic region (Brodeur et al., 2024). The questionnaire included five sections: sociodemographic profile, cannabis and other substance use, Internet use, mental health, and cross-impacts between cannabis and Internet use (Brodeur et al., 2024). For this study, we used data from the sociodemographic, substance use, Internet use, and mental health sections.

### 2.3 Measures and variables

#### 2.3.1 High-risk of cannabis use disorder

The Cannabis Abuse Screening Test (CAST) is widely used in population research to identify individuals at risk of cannabis use disorder (Legleye et al., 2007). It consists of six self-reported items assessing problematic cannabis use, scored on a 0–5 Likert scale. Total scores range from 0 to 24. A score ≥ 7 indicates high-risk of cannabis use disorder and was used as an inclusion criterion in our study (Legleye et al., 2007). The CAST was validated in French and English and demonstrated adequate psychometric properties (Artigaud et al., 2020).

#### 2.3.2 Outcome – depressive symptoms

Depressive symptoms were measured using the Patient Health Questionnaire-8 (PHQ-8) (Kroenke et al., 2009). This self-report tool includes eight items assessing symptom frequency over the past two weeks, scored from 0 (“not at all”) to 3 (“nearly every day”), for a total score of 0–24. For clinical interpretation and analyses, the scores were dichotomized into two categories: minimal to mild (0–9) and moderate to severe (≥10) (Shin et al., 2019). The PHQ-8 was validated in French and English, with strong psychometric properties (Arthurs et al., 2012).

#### 2.3.3 Explanatory variable – Problematic Internet use (PIU)

The Internet Addiction Test (IAT) is widely used to assess PIU (Young & Nabuco de Abreu, 2011). It includes 20 items rated on a 0–5 Likert scale, with total scores ranging from 0–100. The IAT captures key dimensions of PIU such as salience (preoccupation), excessive use, loss of control, anticipation, tolerance, withdrawal-like symptoms, neglect of daily responsibilities, impaired social functioning, and use as a coping strategy for negative emotions. Following common practice, we categorized participants as nonproblematic users (<50) or problematic users (≥50) (Berner et al., 2014; Khazaal et al., 2008; Liberatore et al., 2011; Yen et al., 2009). The IAT was validated in French and English and demonstrated adequate psychometric properties (Moon et al., 2018).

#### 2.3.4 Covariates

Additional variables included sociodemographic characteristics, substance use behaviors, and anxiety symptoms. Substance use was assessed using the Alcohol, Smoking and Substance Involvement Screening Test (ASSIST) (Humeniuk et al., 2008), which screens risk levels across multiple substances, including tobacco, alcohol, cannabis, cocaine, amphetamine-type stimulants, inhalants, sedatives or tranquilizers, hallucinogens, opioids, and other drugs used for nonmedical purposes. Anxiety symptoms were measured using the Generalized Anxiety Disorder-7 (GAD-7) scale (Löwe et al., 2008).

### 2.4 Statistical analyses

Given the secondary nature of the data, as a first step, potential covariates were selected from the extensive dataset based on prior evidence of their association with depressive symptoms in the literature (Remes, Mendes and Templeton, 2021a, Remes, Mendes and Templeton, 2021b) and the clinical expertise of the research team. Bivariate analyses were then conducted to compare independent groups with and without depressive symptoms, using chi-square tests for categorical variables and Mann–Whitney *U* tests for continuous variables. Only variables with a *p* < .05 were retained as covariates for further analysis: age, sex at birth, sexual orientation, marital status, university education level, main occupation, annual pre-tax income, problematic tobacco use, problematic alcohol use, problematic use of other substances, and anxiety symptoms.

For the first objective descriptive analyses were conducted to characterize the sample and subgroups with and without PIU. Categorical variables were summarized as frequencies and percentages; the continuous variable (GAD-7 score) was summarized using medians and interquartile ranges. Group comparisons used chi-square tests for categorical variables and the Mann–Whitney *U* test for continuous variables, as the data did not follow a normal distribution (Fortin & Gagnon, 2022).

For the second objective a multivariate binary logistic regression model was used to estimate the independent effect of PIU on depressive symptoms. Following the rule of thumb of 10 observations per estimated parameter, the number of covariates included was determined to ensure adequate statistical power and to avoid overfitting (Bursac et al., 2008; Harrell et al., 1996). Multicollinearity was assessed using Pearson correlation coefficients (*r* < 0.80) and variance inflation factors (VIF < 5) (James et al., 2013; Shrestha, 2020). A backward stepwise selection method was applied (R. X. Liu et al., 2003). The results were presented as odds ratios (*OR*) with 95% confidence intervals (CI). Analyses were conducted using SPSS version 28.0, with statistical significance set at *p* < .05.

## 3 Results

### 3.1 Sample characteristics

Table 1 summarizes the sample characteristics. Most participants were men (65.4%) aged 18–54 years (85.2%). Over half were single, 16.5% held a university degree, and 70.4% were employed, with 15.1% unemployed. More than half reported an annual income of < CAD$50,000. Problematic substance use was common, particularly tobacco use (61.0%). The median anxiety symptoms score was 5 (IQR: 1–9). Overall, 10.6% met the criteria for PIU, and 26.5% reported moderate to severe depressive symptoms.

**Table 1:** Sample and subgroup characteristics.

**Variables**	**Total** *N* = 874 (100%)	**Without PIU***N* = 781 (100%)	**With PIU***N* = 93 (100%)	***p*-value**
**Sociodemographic Profile**				
Age				
18–34 years	343 (39.2)	278 (35.6)	65 (69.9)	<.001 ^a^
35–54 years	402 (46.0)	374 (47.9)	28 (30.1)	
55+ years	129 (14.8)	129 (16.5)	0 (0.0)	
Sex at birth: Male	572 (65.4)	501 (64.1)	71 (76.3)	.02 ^a^
Sexual orientation: sexual diversity	162 (18.5)	142 (18.2)	20 (21.5)	.43 ^a^
Marital status				
Single	452 (51.7)	398 (51.0)	54 (58.1)	.034 ^a^
Separated/ Widowed	66 (7.6)	65 (8.3)	1 (1.1)	
In a relationship	356 (40.7)	318 (40.7)	38 (40.9)	
Education: University	144 (16.5)	112 (14.3)	32 (34.4)	<.001 ^a^
Main occupation				
Employed	610 (70.4)	536 (69.1)	74 (81.3)	<.001 ^a^
Student	47 (5.4)	34 (4.4)	13 (14.3)	
Retired	79 (9.1)	79 (10.2)	0 (0.0)	
Unemployed	131 (15.1)	127 (16.4)	4 (4.4)	
Annual personal income (before taxes)				
≤ CAD$ 29,999	245 (28.7)	225 (29.6)	20 (21.5)	.01 ^a^
CAD$ 30,000–49,999	230 (26.9)	213 (28.0)	17 (18.3)	
CAD$ 50,000–79,999	220 (25.8)	185 (24.3)	35 (37.6)	
≥ CAD$ 80,000	159 (18.6)	138 (18.1)	21 (22.6)	
**Problematic substance use (ASSIST: Moderate to High Risk)**				
Problematic tobacco use	527 (61.0)	468 (60.4)	59 (66.3)	.28 ^a^
Problematic alcohol use	266 (31.3)	203 (26.6)	63 (74.1)	<.001 ^a^
Problematic use of other substances	160 (18.4)	111 (14.2)	49 (54.4)	<.001 ^a^
**Mental health profile**				
Depressive symptoms (PHQ-8 ≥ 10)	232 (26.5)	178 (22.8)	54 (58.1)	<.001 ^a^
Anxiety symptoms (GAD-7, median [IQR])	5 (1–9)	4 (1–8)	10 (6.9–12)	<.001

Note: Weighted data. Percentages may not total 100% due to rounding. Percentages in parenthesis are relative to the column total. (a) Groups were compared using Chi-square tests. Mann–Whitney U test was used otherwise. Statistical significance set at p < .05 (two-tailed). PIU: Problematic Internet use; PHQ-8: Patient Health Questionnaire – 8 items; ASSIST: Alcohol, Smoking and Substance Involvement Screening Test; GAD-7: Generalized Anxiety Disorder – 7 items; IQR: interquartile range.

### 3.2 Characteristics of participants with and without PIU

Moderate to severe depressive symptoms were significantly more prevalent in the PIU group (58.1% vs. 22.8%, *p* < .001). Regarding other characteristics, individuals with PIU were significantly younger (69.9% aged 18–34 years vs. 35.6% without PIU, *p* < .001). None of the participants with PIU was aged ≥55. Men were more prevalent in the PIU group (76.3% vs. 64.1%, *p* = .02). A higher proportion of PIU participants had a university degree (34.4% vs. 14.3%, *p* < .001). PIU participants were more likely to be employed (81.3% vs. 69.1%) or students (14.3% vs. 4.4%), and none were retired. PIU was also more frequent among those with higher incomes (*p* = .01). Problematic alcohol use (74.1% vs. 26.6%, *p* < .001) and use of other substances—including cocaine, amphetamine-type stimulants, inhalants, sedatives or tranquilizers, hallucinogens, opioids, and other drugs used for nonmedical purposes (54.4% vs. 14.2%, *p* < .001)—were significantly more common in the PIU group. Median anxiety symptom scores were higher among PIU participants (10 vs. 4, *p* < .001).

### 3.3 Statistical modelling of PIU and depressive symptoms

**Fig. 1:**
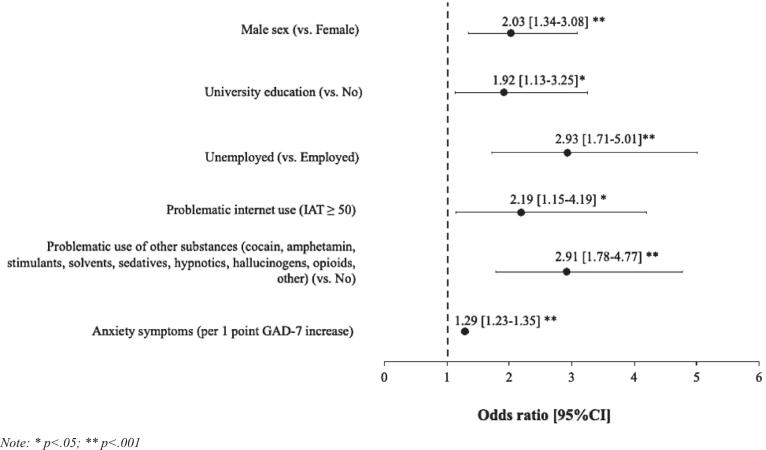
Logistic regression model of the association between PIU and depressive symptoms. Note: * p < .05; ** p < .001.

After controlling for sociodemographic variables, substance use, and mental health indicators, the multivariate binary logistic regression analysis revealed that a significant association remained between PIU and moderate to severe depressive symptoms (*OR* = 2.19; 95% CI: 1.15–4.19; *p* = .018) (Fig. 1).

The final adjusted logistic regression model yielded a Nagelkerke *R*² value of 0.487, indicating satisfactory explanatory power (Nagelkerke, 1991). The model's statistical power was 0.97, reflecting a very high probability of detecting a moderate effect on our variable of interest (Dziak et al., 2020).

## 4 Discussion

This study aimed to examine PIU and its relationship with depressive symptoms among adults at high-risk of cannabis use disorder. Specifically, we sought to describe the characteristics of individuals with and without PIU, focusing on the presence of depressive symptoms, and to assess the association between PIU and depressive symptoms within this specific population.

Our study represents an initial exploration of the link between PIU and depressive symptoms among adults who are recreational cannabis users at high-risk of cannabis use disorder, using a large population-based sample. Recent research has increasingly focused on PIU and mental health disorders. Numerous studies have examined the link between PIU and depressive symptoms in general and specific populations, such as students (Kumar et al., 2024), psychiatric patients (de Vries et al., 2018), and civil air crew members (Zhang et al., 2025). The works cited below, mostly literature reviews, illustrate the growing body of research on this topic: Andrade et al., 2020; Cai et al., 2023; Kumar et al., 2024; Pham et al., 2025; Sánchez-Fernández et al., 2023; Ye et al., 2023 and Zhang et al., 2025. However, to our knowledge, no study has specifically investigated individuals at high-risk of cannabis use disorder.

Our focus on adults was particularly relevant given the limited number of studies on the association between PIU and depressive symptoms conducted in this age group (18 years and older), as most prior research has targeted younger populations, such as adolescents (Pham et al., 2025; Ye et al., 2023) or emerging adults (18–34 years old) (Kumar et al., 2024; Pham et al., 2025; Sánchez-Fernández et al., 2023; Vázquez-Martínez et al., 2024). Moreover, many of these studies were conducted in narrowly defined settings, such as universities (Kumar et al., 2024; Sánchez-Fernández et al., 2023; Vázquez-Martínez et al., 2024), which limits the generalizability of the findings to broader populations.

Because this is the first study on the topic, no direct comparison with similar populations is possible. To contextualize our findings, we drew parallels with data from populations without cannabis use disorder reported in the literature. This approach points to potential similarities and differences between individuals with PIU who are of at high-risk cannabis use disorder and those without such risk. Although these comparisons were not directly tested in this study, they provide a useful frame of reference. Therefore, future research should explore this comparison more systematically.

Among our sample of Quebec adults presenting high-risk of cannabis use disorder, those with concurrent PIU exhibited a higher prevalence of depressive symptoms. This observation is consistent with findings reported in more general adult populations (de Vries et al., 2018; Pohl et al., 2021). However, in our sample, individuals with PIU were more likely to be employed and to report higher income levels. This contrasts with patterns typically observed in more general populations, where individuals with PIU are more frequently unemployed and have lower income (Chen et al., 2025; Faltýnková et al., 2020; Urbanova et al., 2019). Apart from these two variables, other sociodemographic and clinical characteristics were largely consistent with those found in more general populations with PIU (Cai et al., 2023; Ho et al., 2014; Liu et al., 2025; Lopez-Fernandez et al., 2023; Rücker et al., 2015; Sun et al., 2025; Wang et al., 2022; Yang et al., 2024).

In addition, the results indicate that among Quebec adults at high-risk of cannabis use disorder, those exhibiting PIU were approximately twice as likely to report depressive symptoms compared to those without PIU. This link remained statistically significant after controlling for sociodemographic and clinical variables. These results are consistent with previous research conducted in more general populations, where comparable associations have been documented (Pham et al., 2025; Zhang et al., 2025). Drawing on recent conceptual and empirical work (Brand et al., 2026; Remes, Mendes and Templeton, 2021a, Remes, Mendes and Templeton, 2021b), we highlighted common factors frequently linked to both PIU and depressive symptoms, including: emotional distress and mental health factors (e.g., stress/anxiety) and related maladaptive coping; sleep disturbances and poor sleep quality; emotion regulation difficulties; cognitive vulnerabilities (e.g., maladaptive beliefs, cognitive distortions); poor attention control; social factors such as loneliness, reduced social support, and relationship context; and family environment. Taken together, these shared factors may help account for the co-occurrence of PIU and depressive symptoms. Cannabis use may also contribute to this association. Indeed, cannabis use has been linked to several features related to depression, including emotional withdrawal, cognitive impairment, and disruptions in reward processing, all of which may exacerbate depressive symptoms (Langlois et al., 2021). Some studies highlighting the role of internalizing symptoms (e.g., anxiety, depression) as partial mediators between cannabis use and PIU (Bélisle et al., 2025; Doggett et al., 2021). Furthermore, the literature suggests that cannabis use disorder is associated with experiential avoidance (Sequeda et al., 2026). In the context of PIU, experiential avoidance may significantly predict subsequent depressive symptoms, while depression may, in turn, predict subsequent PIU (Cao et al., 2023).

Moreover, although the current literature does not yet clearly establish whether specific online content, platforms, or patterns of Internet use are relatively more important—particularly because few studies have compared these dimensions within the same design—some evidence suggests that problematic forms of digital engagement may be more strongly related to depression than duration of use alone. For example, Internet gaming disorder and problematic smartphone use have both been associated with depressive symptoms (Augner et al., 2023, Ostinelli et al., 2021). Similarly, in the context of social networking sites, problematic use appears more strongly associated with depression than time spent or general use intensity (Cunningham et al., 2021). Potential mechanisms may include upward social comparison, which has been linked to depression and broader psychological maladjustment (Lei et al., 2026; Yoon et al., 2019), as well as cybervictimization, which has been identified as a risk factor for internalizing problems including depression (Marciano et al., 2020).

Finally, the interpretation of these findings should take into account the unique socio-cultural context of Quebec, shaped by factors such as cannabis legalization, consumption habits, prevailing attitudes toward cannabis, social norms, and patterns of Internet use (Ali-Hassan et al., 2019; Andrade et al., 2020; Audy et al., 2021; Djapa & Lapointe, 2025; Hall et al., 2023). Therefore, the observed results may vary across different cultural or geographic settings. The generalizability of these findings is therefore limited to the Quebec context.

### 4.1 Strengths and limitations

This study has two main strengths. First, our sample is representative of adults in Quebec who are recreational cannabis users at high-risk of cannabis use disorder, which increases the external validity of the findings. Second, the use of validated and widely recognized measurement tools also ensures strong internal validity (Fortin & Gagnon, 2022).

Nevertheless, certain limitations must be acknowledged. The data were self-reported and thus subject to potential information bias, such as recall errors or social desirability, which may have affected estimate accuracy. Moreover, the absence of formal clinical diagnoses for PIU or cannabis use disorder could reduce the precision of case identification. The model also does not account for unmeasured confounding variables that were unavailable in the “Cannabis Use, Mental Health, and Problematic Internet Use in Quebec (Canada)” project database (Brodeur et al., 2024), such as social support, environmental factors, lifestyle habits, or the potential effects of COVID-19 on cannabis or Internet use. In addition, multiple statistical comparisons were conducted without applying a correction for multiple testing (e.g., Bonferroni), which may increase the risk of type I error. Moreover, because our data come from a Quebec-based sample, the findings may not generalize to populations in different sociocultural or healthcare contexts. Finally, the cross-sectional design precludes causal inference between PIU and depressive symptoms or the identification of predictive factors.

### 4.2 Implications and impact

Our findings point to potential scientific, clinical, and societal implications. From a scientific perspective, this study advances empirical understanding of the associations among PIU, depressive symptoms, and cannabis use disorder. The findings provide novel insights into how PIU may relate to depressive symptoms within this population and emphasize the need for future research to better clarify the temporal and causal dynamics linking cannabis use disorder, PIU, and depressive symptoms, notably by prioritizing longitudinal designs capable of disentangling directionality and timing of onset. Beyond documenting associations, mechanistic and mediational models are needed to test plausible explanatory pathways—such as emotion dysregulation, coping motives, stress exposure, sleep disruption, and impairments in offline functioning—that may account for the co-occurrence and persistence of these conditions. Intervention studies are also required to evaluate whether targeting shared mechanisms can yield across-domain improvements, and to determine the integration of care for individuals presenting with these comorbid profiles.

Clinically, these findings offer preliminary, evidence-informed insights that may help raise awareness among professionals regarding PIU as a potentially relevant factor associated with depressive symptoms in individuals at high-risk of cannabis use disorder. Such awareness could encourage clinicians to consider early screening for PIU and depressive symptoms in individuals with cannabis use disorder or at high-risk of cannabis use disorder. These considerations may contribute to improving clinical assessment and care strategies by promoting the early identification of comorbid conditions and informing more tailored interventions.

Finally, at the societal level, this study sheds light on the emerging interplay between PIU and depressive symptoms among individuals at high-risk of cannabis use disorder. It emphasizes the relevance of this association for public health and the importance of encouraging increasing awareness of these co-occurring issues. In Quebec, public policy and community-based action have jointly advanced prevention and educational measures aimed at healthier screen use and reduced hyperconnectivity (e.g. Quebec Strategy on Screen Use and Youth Health 2022–2025 (“Stratégie québécoise sur l'utilisation des écrans et la santé des jeunes 2022-2025”) (Ministère de la Santé et des Services sociaux, 2022) and Interministerial Action Plan on Addiction 2018–2028 (“Plan d'action interministériel en dépendance 2018-2028”) (Ministère de la Santé et des Services sociaux, 2022)). These policy frameworks target both youth and adults, highlighting the relevance of deploying prevention efforts, including those related to PIU, in order to reach a broader range of individuals through existing addiction and mental-health service pathways. Also, promoting accessible science communication for a wider audience—through educational videos, digital content, or community-based initiatives—could help disseminate these findings and encourage informed dialogue within the community.

## 5 Conclusion

This study underscores the relevance of PIU as a factor associated with depressive symptoms in adults at high-risk of cannabis use disorder. The findings revealed that individuals with PIU present distinct sociodemographic and psychiatric characteristics compared with those without PIU. Importantly, the association between PIU and depressive symptoms remained significant, even after controlling for other variables. These findings provide initial insights into the relationship between PIU and depressive symptoms among individuals at high-risk of cannabis use disorder within the specific sociocultural context of Quebec (Canada). They also emphasize the importance of further research both in Quebec and across diverse geographical and cultural settings, as well as longitudinal studies, to further understanding and raise awareness of this emerging issue.

## CRediT authorship contribution statement

**Rasolofomamonjy Rasoamiadana Volanirina:** Writing – original draft, Visualization, Project administration, Methodology, Formal analysis, Conceptualization. **Dufour Isabelle:** Writing – review & editing, Validation, Supervision, Methodology, Conceptualization. **Hudon Catherine:** Writing – review & editing, Validation, Supervision, Methodology, Conceptualization. **Carrier Nathalie:** Writing – review & editing, Validation, Formal analysis. **Muñoz Gómez Natalia:** Writing – review & editing, Validation. **Brodeur Magaly:** Writing – original draft, Validation, Supervision, Methodology, Funding acquisition, Conceptualization.

## Funding

This study was supported by the Fonds de recherche du Québec – Santé (FRQS), project number 324497- DOI: 10.69777/324497. The funder had no involvement in the design of the study; data collection, management, analysis, or interpretation; manuscript preparation, review, or approval; or the decision to submit the manuscript for publication.

## Declaration of competing interest

The authors declare that they have no known competing financial interests or personal relationships that could have appeared to influence the work reported in this paper.

## Data Availability

Data will be made available on request.
